# A complicated case of relapsing polychondritis: Case report

**DOI:** 10.1097/MD.0000000000042987

**Published:** 2025-06-20

**Authors:** Shaobo Guo, Longzhao Li, Lu Wang

**Affiliations:** a School of Nursing, Beijing University of Traditional Chinese Medicine, Beijing, China; b Respiratory Disease Center, Dongzhimen Hospital, Beijing University of Chinese Medicine, Beijing, China.

**Keywords:** amyloidosis, bronchoscopy, case report, laser tracheobronchoplasty, relapsing polychondritis

## Abstract

**Rationale:**

Relapsing polychondritis (RP) is a rare inflammatory disease that presents clinically with severe dyspnea and respiratory distress. Differentiating it from amyloidosis can be diagnostically challenging. In cases of severe respiratory distress, timely bronchoscopic intervention, including laser tracheobronchoplasty and glucocorticoids (GCs) spraying, is crucial. Efficacy of this combined approach has not been reported to treat onset of RP.

**Patient concerns:**

We report a complex case of RP in a 56-year-old male. Over the past 2 years, the patient experienced recurrent episodes of dyspnea, cough, and expectoration. Chest computed tomography (CT) scans indicated tracheal wall thickening, and airway stenosis and deformation. Recently, the patient presented with severe dyspnea and respiratory distress, leading to a definitive diagnosis of RP.

**Diagnoses:**

The diagnosis of RP was established based on the patient’s medical history, CT scans, clinical signs (noting cartilage collapse and swelling in the ears, nasal collapse), and endoscopic findings (severe airway stenosis and collapse).

**Interventions:**

The patient was treated with noninvasive ventilation, budesonide, GCs for anti-inflammatory effects, and moxifloxacin for anti-infective therapy. Due to worsening conditions, invasive ventilation was used for 4 days. An emergency bronchoscopic examination was performed, followed by sputum aspiration, laser tracheobronchoplasty for airway reshaping, and endotracheal intubation to stabilize oxygen saturation and alleviate symptoms. Cyclophosphamide was administered.

**Outcomes:**

The patient experienced significant relief from dyspnea, and no recurrence was observed within 1 month after the completion of treatment.

**Lessons:**

When RP is exacerbated by infection, leading to progressive dyspnea and causing acute respiratory distress, it is difficult to distinguish based on CT scans. In situations where pathological results are not promptly available, endoscopic diagnosis and intervention are merited, we recommend performing laser tracheobronchoplasty early during bronchoscopy and spraying GCs to reduce mucosal edema.

## 1. Introduction

Relapsing polychondritis (RP) is a rare inflammatory disease that affects multiple organs, including the eyes, ears, and respiratory tract, and involves the cartilage structures of various systems, such as the tracheobronchial and cardiovascular systems.^[1]^ More critically, RP can lead to inflammation and destruction of tracheal and bronchial cartilage, resulting in airway narrowing, severe dyspnea, and wheezing. In particular, in cases with concurrent infections, patients may experience severe hypoxia, which can be life-threatening. Diagnosis is typically based on the McAdam criteria,^[2]^ and treatment mainly involves the use of glucocorticoids (GCs) and immunosuppressive agents.^[3]^ However, due to the complexity of patient symptoms and the lack of standardized guidelines, treatment approaches remain diverse.^[4]^

In this case report, we present a complex case of RP that was initially misdiagnosed as amyloidosis and incorrectly treated at a local hospital, leading to a delay in effective treatment and the onset of severe dyspnea. After the patient was transferred to our hospital, we confirmed the diagnosis of RP based on his medical history and clinical presentation.

A previous case report by Wu et al^[5]^ described a patient with RP who presented with near-complete central airway collapse and was successfully stabilized using bronchoscopic laser tracheobronchoplasty, stent placement, and corticosteroid therapy. While this case demonstrated the efficacy of bronchial interventions, our case further highlights the critical importance of timing and the applicability of such interventions in patients with acute respiratory distress. For patients with severe respiratory distress, bronchoscopy and intervention can be performed early during hospitalization, without waiting for soft tissue pathology results or successful stent placement. Additionally, we highlight the effectiveness of spraying GCs during laser tracheobronchoplasty to reduce mucosal edema and inflammation, preventing further airway narrowing.

This case report adheres to the CARE reporting guidelines^[6]^ for the presentation of a case study. Historical and current information from this episode of the case is organized as a timeline (Figure S1, Supplemental Digital Content, https://links.lww.com/MD/P251).

## 2. Case report

### 2.1. Patient information

On April 15, 2024, a 56-year-old male patient was transferred to our department due to dyspnea and recurrent infections. The patient had a history of ocular inflammation, hypertension for 4 years, and kidney stones for 2 years. He did not have a history of diabetes, heart disease, and other hepatic or renal diseases.

Two years prior to admission, the patient was treated at a local hospital for cough and dyspnea. Computed tomography (CT) scans showed apparent bilateral lung infections and bronchial inflammatory changes with stenosis. He was treated with anti-inflammatory, anti-infective, and antitussive therapies. Over the next 2 years, he experienced recurrent episodes of dyspnea, cough, expectoration, and fever.

Two weeks before transfer, the patient received standard anti-inflammatory and anti-infective treatment at a local hospital, including intravenous infusion of amoxicillin 500 mg twice daily (bid) and lysine aspirin 0.9 g intravenous bolus once daily (qd), but the symptoms did not improve. Considering the patient’s history of chronic cough and airway stenosis, amyloidosis was suspected. Despite receiving oxygen therapy, routine anti-inflammatory treatment, and low-dose GCs at the local hospital, his condition deteriorated, leading to a referral to our department.

### 2.2. Diagnostic assessment

**Figure 1.**
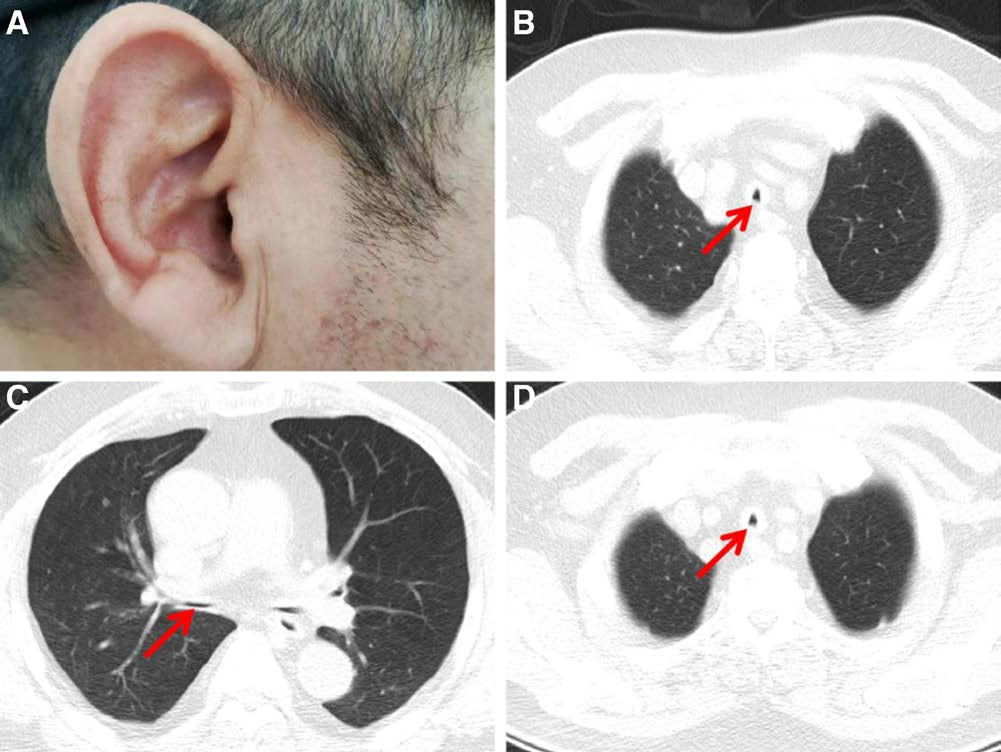
(A) Onset of cartilage collapse and swelling in the patient’s ears. (B) CT plain scan shows diffuse thickening of the tracheal and bronchial walls, with the red arrow indicating the airway wall thickening. (C) CT plain scan identifies airway stenosis and collapse, with the red arrow signifying the main airway collapse. (D) The red arrow points the bronchial collapse. CT = computed tomography.

Physical examination revealed cartilage collapse and swelling in the patient’s ears (Fig. 1A), conjunctival congestion and tearing, significant nasal collapse, and bilateral inspiratory and expiratory wheezing in the upper lobes of the lungs. The patient’s oxygen saturation was 95%, and according to the Modified British Medical Research Council Questionnaire dyspnea scale,^[7]^ the patient’s score for respiratory distress was 3. The emergency chest CT showed significant diffuse thickening of the tracheal walls, as well as airway stenosis and collapse (Fig. 1B–D).

### 2.3. Therapeutic intervention

The patient was in severe respiratory distress upon transfer, unable to move independently, with oxygen saturation equal to 90%. Due to the respiratory distress, we utilized a noninvasive ventilator (ventilator mode ST, IPAP: 12 cm H_2_O, EPAP: 6 cm H_2_O). We administered budesonide inhalation (1 mg) 3 times daily (tid), along with intravenous doxophylline (0.3 g) tid and moxifloxacin (0.4 g) qd, to relax smooth muscles, reduce inflammation, and combat infection. After using noninvasive ventilation and anti-infection and anti-asthma treatments, the patient still experienced severe dyspnea, with SpO_2_ of 93%. Because of severe airway collapse, the patient continued to experience wheezing and unstable oxygen saturation after treatment. Given the significant airway collapse, combined with the excessive area of stenosis in the central airway of the patient, we recommended placement of a Y-shaped stent to support the airway and maintain ventilation. However, due to financial concerns, the patient firmly refused the insertion of a stent.

**Figure 2.**
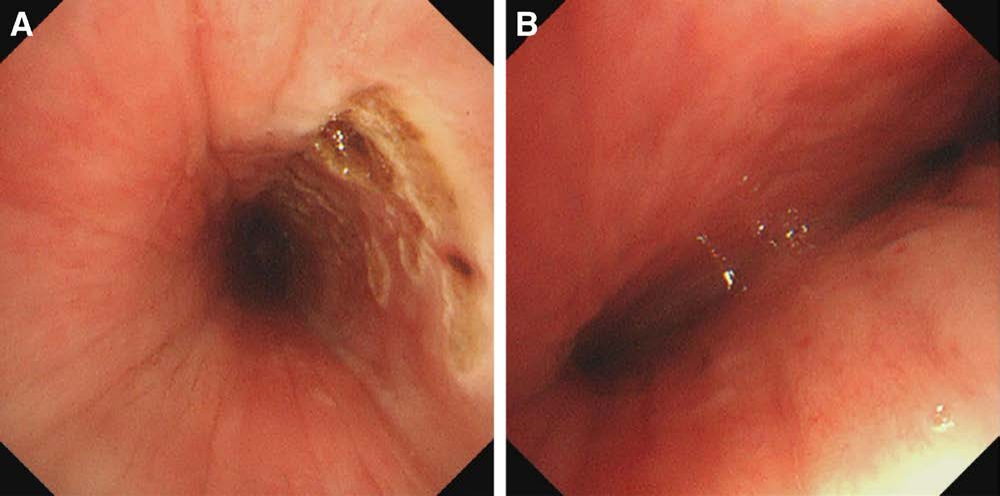
Bronchoscopic findings in the patient. (A) It reveals significant softening and the presence of viscous secretions. (B) It shows airway collapse, stenosis, and a large amount of secretions. CT = computed tomography.

On the second day of admission, the patient’s oxygen saturation could not be maintained and dropped to 90%. We then performed an emergency bronchoscopic examination, which revealed significant softening, collapse, and stenosis of the patient’s left and right main bronchi, with a large amount of viscous secretions in the lumen (Fig. 2A,B). We suctioned excessive mucus from the affected areas, followed by laser tracheobronchoplasty for airway remodeling. Additionally, 5 mg of GCs was sprayed locally via bronchoscopy to reduce mucosal edema. Subsequently, we conducted an emergency endotracheal intubation (6.5#), with the tip positioned 24 cm from the edge of the patient’s incisors. The invasive ventilator was set to PC mode with an oxygen concentration of 40%, PEEP 8 cmH_2_O, and a frequency of 17 breaths/min, which restored oxygen saturation to 100% and alleviated the patient’s dyspnea.

We conducted a comprehensive analysis of the patient’s condition, noting cartilage collapse and swelling in the ears, significant nasal collapse, and severe airway stenosis and collapse observed under bronchoscopy. The patient also presented with conjunctival injection and epiphora, and a thorough review of the patient’s medical history revealed a history of bilateral chronic panuveitis. In accordance with the McAdam^[2]^ criteria for RP, the patient exhibited the characteristic manifestations of the disease in the ears, nose, and airways, and also had ocular involvement, leading to a diagnosis of RP. We then administered intravenous methylprednisolone sodium succinate 160 mg bid and provided nutritional and airway management. For nutritional support, the patient remained nil per os and administered 500 mL of enteral nutrition emulsion (TPF-T) bid via nasogastric tube. For airway management, we adjusted the mode, flow, and duration of oxygen therapy based on blood gas analysis at any time, utilized noninvasive ventilation as needed, performed aerosol inhalation with budesonide (1 mg) tid for viscous sputum, and trained the patient bid to perform abdominal breathing and lip contraction exercises.

### 2.4. Outcomes

On the fifth day of admission, the patient’s dyspnea improved, and he no longer experienced severe shortness of breath. Their temperature remained stable, and blood gas analysis results were nearly normal, with oxygen saturation maintained above 95%. As a result, we extubated the patient and transitioned to 2 L/min nasal cannula oxygen. A low-sodium, low-fat diet was initiated thereafter. By the ninth day of admission, the patient’s symptoms had partially improved, with a reduction in cough and sputum production. Oxygen saturation returned to over 98%, at which point oral cyclophosphamide (CTX) was started at a dosage of 100 mg qd.

On the 12th day of admission, as the symptoms were relieved, methylprednisolone was changed to an oral dose of 40 mg qd. The patient was discharged on day 14 and prescribed a daily regimen of 30 mg methylprednisolone and 50 mg CTX. During the 1-month follow-up, the patient reported a good recovery with no recurrence of dyspnea.

## 3. Discussion

RP is a rare immunologically related disease, with an annual incidence rate of approximately 0.7 to 9.0 cases per million people.^[3]^ The pathogenesis of RP remains unclear, and antibodies specific to cartilage proteins, such as antitype II collagen and anti-matrilin-1, lack sensitivity and specificity. Therefore, the diagnosis of RP largely relies on the description of clinical features such as local inflammation in areas like the ears, joints, and nasal passages, with respiratory tract involvement being relatively less common (21–56%).^[8–10]^ However, the impact of RP on systemic immunity results in significant variability in clinical manifestations among patients. Those with atypical symptoms often suffer from misdiagnosis and delays in treatment, posing a significant management challenge for clinicians.^[11]^

This patient exhibited symptoms of airway stenosis, cough, shortness of breath, and dyspnea, with symptoms exacerbated by a history of infection-induced respiratory distress, which resembled amyloidosis and could not be distinguished based solely on CT scans.^[12,13]^ Therefore, a diagnosis should integrate clinical findings from other affected organs and the patient’s medical history. This patient exhibited symptoms of auricular and nasal chondritis and had a history of bilateral chronic panuveitis. The McAdam^[2]^ criteria indicate that RP can be diagnosed with the presence of 3 or more symptoms from 6 chondritis areas (ears, nose, eyes, joints, respiratory tract, hearing, or vestibular dysfunction). The patient exhibited the characteristic manifestations of the disease in the ears, nose, and airways, and also had ocular involvement, leading to a diagnosis of RP.

However, in this case, the patient experienced severe dyspnea due to the combined effects of the infection-induced respiratory distress of RP and prior incorrect treatment. This symptom was pronounced and critical, and oxygen saturation could not be easily maintained despite pharmacological treatment. It was clearly impractical to wait for a cartilage biopsy to further confirm the diagnosis of RP. Therefore, observation and intervention under bronchoscopy were deemed necessary. Firstly, bronchoscopic examination revealed significant airway collapse and deformation, aiding in the diagnosis of RP. Secondly, bronchoscopic suctioning of secretions and endotracheal intubation restored the patient’s oxygenation and airway patency, which provided an opportunity for subsequent high-dose steroid pulse therapy and the use of CTX. Furthermore, we propose that early intervention with laser tracheobronchoplasty^[14]^ for airway remodeling, combined with localized GCs administration to reduce mucosal edema, may offer substantial clinical benefits in alleviating dyspnea. This approach could be initiated during the initial bronchoscopy.

In this case, due to the patient’s severe dyspnea and significant airway collapse, we performed an emergency bronchoscopic examination and treatment, followed by timely endotracheal intubation, which ultimately restored oxygenation and airway patency.

## 4. Conclusion

This case demonstrates that during episodes of worsened respiratory distress due to infection, distinguishing RP from amyloidosis is difficult based solely on symptoms and CT scans.^[15,16]^ Especially in the presence of infection, timely differentiation relies on a thorough medical history and prompt bronchoscopy. The previous study^[17]^ has demonstrated the effectiveness of stent placement for RP, showing significant improvement in relieving dyspnea. This case places greater emphasis on the timing of bronchial intervention and its relevance for patients in respiratory distress. Even in the absence of stent placement, early direct bronchoscopy treatment can effectively address respiratory distress in critically ill patients.

Bronchial interventions and tissue sampling can be performed simultaneously during the same procedure. Especially in patients with tracheal collapse, there is no need to wait for definitive soft tissue pathology results or the fabrication of a customized stent. Furthermore, we also demonstrate that early laser tracheobronchoplasty can promote airway remodeling. When combined with GCs spraying, it helps prevent further airway narrowing and reduces inflammation, effectively relieves dyspnea. However, due to the limitations of case reports, the long-term efficacy and safety of early intervention combined with GC usage in this case still require further observation.

## Acknowledgments

We are also grateful to all the researchers, including the physicians, nurses, and technicians, who participated in this study.

## Author contributions

**Conceptualization:** Shaobo Guo.

**Data curation:** Longzhao Li.

**Funding acquisition:** Lu Wang.

**Investigation:** Longzhao Li.

**Supervision:** Lu Wang.

**Writing – review & editing:** Longzhao Li.

## Supplementary Material


