# Supplementary figures and images for: A complicated case of relapsing polychondritis: Case report
Source: Medicine (Baltimore). 2025 Jun 20;104(25):e42987. doi: 10.1097/MD.0000000000042987 (PMC12187260; doi:10.1097/MD.0000000000042987)

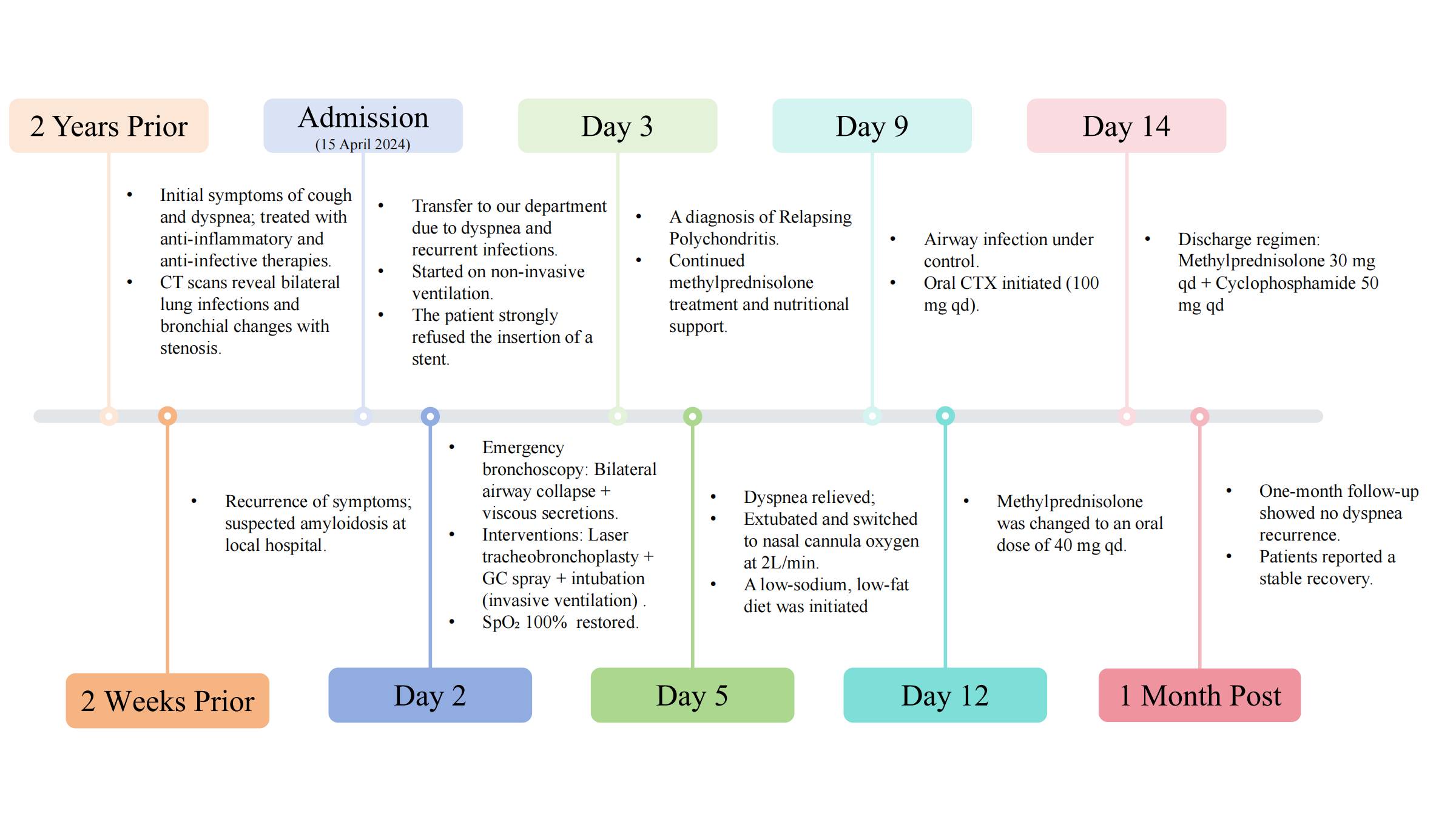

Supplement: Supplementary file 1 [file medi-104-e42987-s001.jpg]
